# Supplementary material for: DNA methylation differences stratified by normalized fetal/placental weight ratios suggest neurodevelopmental deficits in neonates with congenital heart disease
Source: PLoS One. 2025 Aug 6;20(8):e0317944. doi: 10.1371/journal.pone.0317944 (PMC12327636; doi:10.1371/journal.pone.0317944)
Supplement: S1 Table — (PDF) [file pone.0317944.s001.pdf]

S1 Table. Significant Genes Driving Enrichment for Each Gene Pathway

	Ratio Group A vs D	Ratio Group A vs E	Ratio Group D vs E
**Autism SPARK**	ACTB, DNMT3A, ADNP, DSCAM ADSL, DYNC1H1, DYRK1A, AHDC1, EBF3, ALDH5A1, EHMT1, ANK2, EIF3F, ANK3 ANKRD11, FOXG1, ARID1B FOXP1, GIGYF1, ASH1L, GRIN1 GRIN2A, GRIN2B, AUTS2, HIVEP2, BCKDK, BCL11A, BRAF, HRAS, BRSK2, CACNA1C KANSL1, KCNB1, CASZ1, KCNQ3, CBL, KDM3B, KDM6B, CHAMP1, CHD2, CHD3, CHD7, KRAS, CHD8, LZTR1, CIC, CNOT3, MAP2K1, CREBBP MAP2K2, CSDE1, MBD5, CTCF MBOAT7, CTNNB1, CUL3, MED13, MED13L, DEAF1, MEF2C, DHCR7, MEIS2, DLG4, MTOR, DMPK, MYT1L, NAA15, NBEA, NCKAP1, NF1, NIPBL, NLGN2, NR4A2, NRAS, NRXN1, NRXN2, NSD1, PACS1, PHF21A, PHF3, PHIP, POGZ, POMGNT1, PPP1CB, PPP2R5D PTEN, RAF1, RAI1, RALGAPB, RELN, RERE, RFX3, RIT1, SCN2A, SCN8A, SETBP1, SETD2, SETD5, SHANK2, SHOC2, SIN3A, SMARCC2 SON, SOS1, SOS2, SOX5, SPAST, SRCAP, STXBP1, SYNGAP1, TAOK1, TBCK, TBR1, TCF4, TCF20, TRIO, TRIP12, TSC2, UBE3A, VPS13B, WAC, WDFY3, YY1, ZBTB20, ZNF292, ZNF462, MEG3	ACTB, DNMT3A, ADSL, DYNC1H1, DYRK1A, AHDC1, EBF3, EHMT1, ANK2, EIF3F, ANK3, ANKRD11, FOXG1, ARID1B, FOXP1, GIGYF1, ASH1L, GRIN1, GRIN2A, GRIN2B, AUTS2, HIVEP2, BCL11A, HNRNPU, HRAS, BRSK2, CACNA1C, CAPRIN1, KANSL1, KCNB1, CASZ1, KCNQ3, CBL, KDM3B, CHAMP1, CHD2, CHD3, CHD7, KRAS, CHD8, LZTR1, CIC, MAGEL2, MAP2K1, CREBBP, MAP2K2, CSDE1, MBD5, CTCF, MBOAT7, CTNNB1, MED13L, DEAF1, MEF2C, DHCR7, MEIS2, DLG4, MTOR, DMPK, MYT1L, NAA15, NBEA, NF1, NIPBL, NLGN2, NRAS, NRXN1, NRXN2, NSD1, PACS1, PHF21A, PHF3, PHIP, POGZ, POMGNT1, PPP1CB, PPP2R5D, PSMD12, PTEN, RAF1, RAI1, RALGAPB, RELN, RERE, RFX3, SCN8A, SETBP1, SETD2, SETD5, SHANK2, SHOC2, SIN3A, SLC6A1, SON, SOS1, SOS2, SPAST, SRCAP, SYNGAP1, TANC2, TAOK1, TBCK, TBR1, TCF4, TCF20, TRIO, TRIP12, TSC2, UBE3A, VPS13B, WAC, WDFY3, YY1, ZBTB20, ZNF292, ZNF462, MEG3	ACTB, DNMT3A, ADNP, DYNC1H1, AHDC1, EBF3, ALDH5A1, EHMT1, ANK2, ANK3, EP300, ANKRD11, ARID1B, FOXP1, GIGYF1, ASH1L, GRIN1, GRIN2A, GRIN2B, AUTS2, HIVEP2, BCL11A, HNRNPU, BRSK2, CACNA1C, IRF2BPL, KANSL1, KCNB1, CASZ1, KDM3B, KDM6B, CHD2, CHD3, CHD7, KRAS, CHD8, LZTR1, CIC, CREBBP, MAP2K2, MBD5, MBOAT7, CTNNB1, CUL3, MED13L, DEAF1, MEF2C, MEIS2, DMPK, MYT1L, NBEA, NCKAP1, NF1, NIPBL, NLGN2, NR4A2, NRXN1, NRXN2, NSD1, PACS1, PHF21A, PHF3, POMGNT1, PSMD12, PTEN, RAF1, RAI1, RALGAPB, RELN, RERE, RFX3, RORB, SCN8A, SETBP1, SETD2, SETD5, SHANK2, SHOC2, SIN3A, SLC6A1, SMARCC2, SON, SOS1, SOS2, SOX5, STXBP1, SYNGAP1, TAOK1, TCF4, TCF20, TRIO, TRIP12, TSC1, TSC2, UBE3A, VPS13B, WAC, WDFY3, ZBTB20, ZNF292, ZNF462, MEG3
**DisGeNET ADHD**	AS3MT, SLC6A3, MED13, DRD2, CIC, CHRNA4, GRM7, CNR1, PTPRD, CALY, ZNF292, GRM1, DHDDS, CHRNA7, GRIN2A, ASTN2, ST3GAL3, FOXP2, GJB2, PTEN, CACNA1C, PTPRG, NTRK2, SLITRK1, CPLX2, PRKG1, THRB, FAS, AUTS2, PAH, PIK3CA, GABRB3, MITF, GNAS, MLH1, ARVCF, MSH2, SCAPER, MLH3, NTM, RSRC1, WDPCP, MLXIPL,PCNT, RERE, NBN, NCL, NDN, IGF1, ITGAE, STT3A, ITPR3, KCNA2, KCNB1, TUBB2B, HDC, ZNRD1, TAX1BP3, HIVEP1, HLA-DMB, HSPG2, SNORD115-1, ARF1, MIR137HG, SCN8A, EPCAM, ZNF615, LAMA1, MAN2A2, MANBA, SLC13A5, LIMK1, ANK3, MSH6, KRAS, SFTA2, GRIA4, LIG4, MEF2C, STXBP1, PCGF2, TRIM26, CACNA1A, CACNA1B, CACNB2, UBA5, PANK2, SEMA6D, YWHAG, ADAM12, TBX1, TCF4, TCF20, TRIO, TSC2, HIRA, UBE3A, CLIP2, CSRNP3, AP3B2, MLIP,	SLC6A3, TPH2, DRD2, CIC, CHRNA4, GRM7, TACR1, PTPRD, ZNF292, CHRNB2, DHDDS, GRIN2A, ASTN2, ST3GAL3, FOXP2, GJB2, PTEN, CACNA1C, PTPRG, SLITRK1, CPLX2, PRKG1, THRB, AUTS2, PAH, PIK3CA, GABRB3, MITF, GNAS, MLH1, ARVCF, MSH2, GNAQ, SCAPER, MLH3, NTM, RSRC1, WDPCP, MLXIPL, PCNT, RERE, NBN, NCL, NDN, HCN1, ITGAE, STT3A, ITPR3, KCNA2, KCNB1, TUBB2B, HDC, ZNRD1, TAX1BP3, HLA-DMB, HSPG2, HTR2A, ARF1, MIR137HG, SCN8A, EPCAM, ZNF615, LAMA1, MAN2A2, MANBA, SLC13A5, LIMK1, ANK3, MSH6, KRAS, GRIN2D, CEP85L, LHCGR, GRIA4, MEF2C, MKRN3, PCGF2, TRIM26, CACNA1A, CACNB2, SPAG16, UBA5, PANK2, SEMA6D, TBX1, TCF4, TCF20, TGFBR2, TRIO, TSC2, UBE3A, CLIP2, AP3B2, CHAF1B, MLIP, SMC3, GTF2IRD1, SEC24C, FEZ1, SEMA3E, HDAC4, ATP2C2, BAZ1B, BRSK2,	COMT, AS3MT, SLC6A3, TPH2, DRD2, CIC, GRM7, CNR1, GRM8, PTPRD, CALY, ZNF292, GRM1, DHDDS, GRIN2A, ASTN2, ST3GAL3, FOXP2, GJB2, PTEN, CACNA1C, ITIH3, PTPRG, NTRK2, CPLX2, PRKG1, THRB, FAS, AUTS2, PAH, PIK3CA, GABRB3, MITF, GNAS, MLH1, GNAQ, SCAPER, NTM, RSRC1, WDPCP, MLXIPL, PCNT, RERE, NBN, NCL, NDN, ITGAE, STT3A, KCNB1, GTF2I, KIF7, ZNRD1, TAX1BP3, HSPG2, HTR1B, HTR2A, SNORD115-1, KIF5B, MIR137HG, SCN8A, EPCAM, MANBA, SLC13A5, LIMK1, ANK3, MSH6, KIF11, KRAS, GRIN2D, GRIA4, LIG4, MEF2C, STXBP1, TRIM26, CACNA1A, CACNA1B, CACNB2, SPAG16, PANK2, YWHAG, TBX1, TCF4, TCF20, TGFBR2, TRIO, TSC1, TSC2, HIRA, UBE3A, CLIP2, CSRNP3, AP3B2, CHAF1B, MLIP, SMC3, ALKBH8, GTF2IRD1, SEC24C, FEZ1, HDAC4, ATP2C2, BAZ1B, BRSK2, ASCC2, JRK, SYNGAP1,

	SMC3, GTF2IRD1, SEC24C, FEZ1, HDAC4, NUAK1, ATP2C2, KIF14, BAZ1B, BRSK2, FERMT3, ASCC2, PPM1D, JRK, SYNGAP1, SYNJ1, CACNA1H, HERC2, WAC, PPP3CA, PI4K2B, DHTKD1, CHD7, MCTP2, PRKCG, MAPK1, PRNP, C12orf4, TBC1D24, SETD5, ACTL6B, WWOX, ATP6V1A, PMS2, POLG, RBFOX1, CNNM2, DYM, NSUN2, PHIP, SORCS2, PTPRF, RAD21, ARV1, SLC1A2, SLC2A1, SLC6A2, SLC6A9, BMPR1A, SMPD1, SNAP25, SNRPN, SOX5, SPG7, LINC00461, CSMD1, RFC2, RPS20, RREB1, RXRG, BDNF, PIEZO2, BRCA2, FBXL16, DYNC1I2, DYNC1H1, DNM1, DHCR7, ADRA2A, TRAK1, FAN1, CPLX1, NLGN1, GABRA1, JMJD1C, DEAF1, FGF12, PUF60, CHD2, CSMD2, SHANK2, FLII, VPS13A, NUS1, ADNP, SATB2, MED13L, SZT2, SYNE1, FLI1, AK8, NT5C2, C12orf57, SORCS3, ZFPM2, VPS13B, SPRED1, GATA4, CREBBP, SETBP1, CEP112, EMP2, ADCY2, COL2A1, NECAP1, ELN, GNE, IRAK1BP1 CYFIP2, SNORD116-1, ARID2, COL11A2, TBL2, RAI1, CLTC, DPP6, GABRG2, GABRB2, NOP56, MTFMT, SH2B1, EEF1A2, SIN3A, GNB5, ASPM, CRKL, NIPBL, DRD1, CDH13, ELK3, CYP2D6, CLOCK, NTF3, SLC9A9, ADRA1A, EPHB1, WASF2, NGF, CHPT1, BAIAP2, NET1, REM1, GRIN2B, EDNRA, NR3C1, DRD3, NOS1, LPAR2, DDC, ADRA2C, ADRA2B, AMPH, ADIPOQ, VAMP2, SLC1A3, DIRAS2, ZNF804A, STUB1, NR4A2, DISC1, CHRNA3, NCAM1, NANS, TAL1, SYT1, TSPAN31, PIK3CG, ADORA2A, RFC1, POMC, POLR3A, DCDC2, TERF1, BTBD9, TRH, TPO, SCLY, SOD1, TERC, DNMT3B, NDRG2, INTS8, CDK5, SYN3, INSRR, GAD1, DTNBP1, CNTNAP2, BAG3, GRIA1, MTR, ATXN1, IL5, F2R, IL6, DLGAP1, SEC23IP, CDH11, SLC18A2, MTA2, CHRNB3, PKMYT1, NDUFAF2, CHRNB4, SNCA, SLC16A1, SLC39A8, SYT2, CARTPT, SDHD, RBM45, CCL11, CD79A, BHMT, SLC12A6, CNTN1, NSD1, TGM5, NRXN1, FADS2, ITM2B, CD38, DCD, SKI, BCAR1, CLTA, TMX2-CTNND1, VIPR2, ZBBX, MICALL2, RNF122, CIB1, KCNIP4, LMAN2L, CASP3, TNR, TSPAN8, TRIM31, THRSP, MBOAT7,	FERMT3, ASCC2, PPM1D, JRK, SYNGAP1, CACNA1H, HERC2, WAC, PPP3CA, PI4K2B, CHD7, MCTP2, PRKCG, MAPK1, PRNP, SLC4A10, TBC1D24, SETD5, ACTL6B, WWOX, PMS1, POLG, MAGEL2, RBFOX1, CNNM2, DYM, NSUN2, PHIP, SORCS2, PTPRF, ARV1, SLC1A2, SLC6A4, SLC6A9, BMPR1A, SMPD1, SNAP25, SNRPN, SPG7, LINC00461, CSMD1, RFC2, RREB1, BDNF, SDHA, PIEZO2, SEMA4A, BRCA2, PIWIL4, CSNK2A1, GPC6, DYNC1I2, DYNC1H1, DNM1, DHCR7, ADRA2A, TRAK1, FAN1, CPLX1, NLGN1, GABRA1, JMJD1C, DEAF1, FGF12, ITGA11, PUF60, CHD2, CSMD2, SHANK2, FLII, VPS13A, NUS1, SATB2, MED13L, SZT2, SYNE1, FLI1, AK8, NT5C2, SORCS3, ZFPM2, VPS13B, SPRED1, GATA4, CREBBP, SETBP1, EMP2, ADCY2, COL2A1, NECAP1, ELN, GNE, IRAK1BP1, CYFIP2, SNORD116-1, ARID2, COL11A2, TBL2, RAI1, CLTC, DPP6, GABRG2, GABRB2, SH2B1, SIN3AGNB5, CRKL, NIPBL, DRD1, CDH13, ELK3, CLOCK, STX1A, NTF3, SLC9A9, ADRA1A, EPHB1, WASF2, CHPT1, BAIAP2, NET1, REM1, GRIN2B, EDNRA, NR3C1, DRD3, NOS1, NPY, DDC, ADRA2B, AMPH, ADIPOQ, VAMP2, SLC1A3, DIRAS2, SHBG, ZNF804A, STUB1, VEGFA, DISC1, CHRNA3, OXTR, CRP, NANS, TAL1, SYT1, TSPAN31, PIK3CG, ADORA2A, POMC, POLR3A, DCDC2, TERF1, BTBD9, TRH, TPO, CHRNA5, SOD1, TERC, NDRG2, CDK5, SYN3, INSRR, INS, RBM12, GAD1, PER2, CAT, KRT7, DCLK1, CNTNAP2, GRIA1, MTR, ATXN1, IL5, DLGAP1, SEC23IP, ARTN, CDH11, SLC6A1, LGI1, SLC39A13, PDLIM1, PKMYT1, NDUFAF2, SLC16A1, SLC39A8, SYT2, CARTPT, SDHD, RBM45, CCL11, CD79A, BHMT, SCT, SLC12A6, CNTN1, NSD1, NRXN1, FADS2, ADAMTS2, SKI, BCAR1, CLTA, VIPR2, ZBBX, MICALL2, RNF122, KCNIP4, CACNG2, LMAN2L, TNR, TSPAN8, TRIM31, THRSP, MBOAT7, NRG3, VDR, PDE10A, TXN, ZMYM2, TWIST1, SH2B2, RAB40B, CLEC19A, FTO, PHLDA2, THRA, GPHN, SULT2A1, NRP1, STXBP3, STAT6, ARHGEF7, SST, HRH3, PARK7, LIN7A, THBS3, NTPCR, ZGPAT, TH, PSMD14, TSHZ1, BHLHE40, USO1, B4GALT2, NUDT3, CBS, NISCH, KALRN, LAIR1, LAMB2,	SYNJ1, WAC, PPP3CA, PI4K2B, DHTKD1, CHD7, MCTP2, PRKCG, MAPK1, PRNP, SLC4A10, TBC1D24, SETD5, MPP6, WWOX, PMS1, PMS2, POLG, RBFOX1, SORCS2, PTPRF, RAD21, ARV1, SIM1, SLC1A2, SLC2A1, SLC6A2, BMPR1A, SMPD1, SNAP25, SNRPN, SOX5, SPG7, LINC00461, CSMD1, RFC2, RPS20, RREB1, RXRG, BDNF, SDHA, SEMA4A, BRCA2, PIWIL4, CSNK2A1, FBXL16, DYNC1I2, DYNC1H1, ADRA2A, TRAK1, CPLX1, NLGN1, GABRA1, JMJD1C, DEAF1, FGF12, PUF60, CHD2, CSMD2, SHANK2, VPS13A, ADNP, SATB2, MED13L, SZT2, SYNE1, FLI1, CDK20, NT5C2, ZFPM2, VPS13B, SPRED1, GATA4, CREBBP, SETBP1, CEP112, ADCY2, NECAP1, IRAK1BP1, CYFIP2, OSR1, COL11A2, RAI1, DPP6, GABRG2, GABRB2, SH2B1, EEF1A2, SIN3A, GNB5, ASPM, CRKL, NIPBL, CDH13, CLOCK, STX1A, NTF3, SLC9A9, ADRA1A, EPHB1, NGF, CHPT1, BAIAP2, REM1, GRIN2B, NR3C1, NOS1, LPAR2, DDC, ADRA2B, AMPH, ANKK1, SLC1A3, DIRAS2, IMPACT, STUB1, NR4A2, DISC1, NCAM1, OXTR, CRP, BCHE, TAL1, PIK3CG, ADORA2A, POMC, POLR3A, BTBD9, TPO, CHRNA5, SCLY, SPN, SOD1, TERC, NDRG2, SLC2A3, SYN3, GAD1, PER2, DCLK1, NPSR1, DTNBP1, FLRT3, CNTNAP2, BAG3, GRIA1, MTR, ATXN1, F2R, DLGAP1, SEC23IP, CDH11, SLC18A2, SLC6A1, LGI1, SLC39A13, PDLIM1, PKMYT1, NDUFAF2, CHRNB4, SLC16A1, SLC39A8, RBM45, SLC12A6, CNTN1, NSD1, PDLIM7, TGM5, NRXN1, FADS2, THEM4, ADAMTS2, CD38, SKI, BCAR1, CLTA, CNTFR, VIPR2, ZBBX, MICALL2, CIB1, KCNIP4, LMAN2L, CASP3, TNR, TSPAN8, TRIM31, THRSP, MBOAT7, WASF3, NRG3, VDR, PDE10A, TXN, ZMYM2, TWIST1, SH2B2, RAB40B, CLEC19A, FTO, PHLDA2, GPHN, NRP1, STAT6, ARHGEF7, HRH3, RASGRP1, PARK7, NRP2, THBS3, NTPCR, ZGPAT, TH, TSHZ1, BHLHE40, CBS, NISCH, KALRN, FEZF1, LAMB2, LAMC2, LGALS3, FADS1, LMNA, FKBP5, IL16, ITGA1, FN1, ITPA, SYNM, KCNC1, KCNJ5, KCNJ6, CYFIP1, LMX1B, LRP5, MOG, MPP2, MAP1B, ARNTL, MIR138-1, MXD1, FHIT, AMH, GALR1, GUCY2D, NPNT, HFE, KCNIP1, NT5C, GFI1, ZNF544, HTRA2, GCH1, PRTG, GRIA2,

	WASF3, NRG3, VDR, PDE10A, ZMYM2, TWIST1, SH2B2, RAB40B, CLEC19A, FTO, PHLDA2, THRA, GPHN, SULT2A1, NRP1, STXBP3, STATH, STAT6, ARHGEF7, SST, HRH3, RASGRP1, PARK7, NRP2, LIN7A, NTPCR, ZGPAT, TH, PSMD14, TSHZ1, BHLHE40, CBS, NISCH, KALRN, FEZF1, TRIM32, LAMB2, LAMC2, ELFN1, LGALS3, FADS1, LMNA, FKBP5, IL16, ITGA1, FN1, SYNM, KCNC1, RRS1, KCNJ5, KCNJ6, KCNJ11, CYFIP1, LMX1B, LRP6, LRP5, ALDH2, MIP, ALB, NR3C2, MOG, MPP2, MAP1B, ARNTL, MIR320A, MXD1, FHIT, FGF2, GALR1, GSK3B, PADI1, GUCY2D, NPNT, HFE, KCNIP1, GFI1, ZNF544, HTRA2, GCH1, PRTG, GRIA2, FBXO33, HK1, ACACA, COL6A4P1, CADM1, SMUG1, OTP, IGF2, IGFALS, KCNH3, APP, KLK3, HTR6, HRC, ABCA4, HTR3A, HTR5A, P4HTM, PRKCD, ADRA1B, ASH1L, PROC, RELN, PSD, PRKCB, CTNND1, TRIT1, TTC12, CYP2B6, LXN, PTPRN2, PEX2, RAB3A, RARA, CREM, CPT2, ADM, BPIFA2, CRY1, PTGDS, CSE1L, KIDINS220, LRRC7, DPP10, PTPRC, ATXN7, ERBB2, ATM, NF1, NFKB1, NGFR, NTRK1, NTRK3, OPRM1, MYT1, MSMB, C9orf72, EIF4EBP1, EGF, MYO5B, SPOCK3, PC, SERPINA1, PIK3CB, SERPINF2, CNTN5, DAPK3, PON1, CCHCR1, GPRC5B, ASCC1, AGA, GAL, DLX4, DLG2, IPO11, PLA1A, PDE2A, PDE4A, PDE4D, OXER1, DGKH	ELFN1, LGALS3, FADS1, LMNA, FKBP5, IL16, ITGA1, KCNC1, RRS1, KCNJ5, CYFIP1, LMX1B, LRP6, LRP5, FAAH, MFGE8, MIP, ALB, MNT, AKT1, MPP2, MARK1, MAP1B, ARNTL, MIR107, MIR320A, MXD1, FHIT, AMH, GALR1, GSK3B, GUCY2D, HFE, KCNIP1, NT5C, CNTN6, GFI1, ZNF544, HTRA2, PRTG, GRIA2, ACACA, NAT8L, CADM1, SMUG1, OTP, IGF2, APP, KLK3, FRRS1L, HTR6, PRPF6, HRC, ABCA4, HTR1D, HTR1E, HTR3A, HTR4, HTR5A, P4HTM, ASH1L, PSPN, RELN, PSD, PRKCB, CTNND1, PPIA, TRIT1, TTC12, ADRB2, SLC30A10, PEX2, RAB3A, RARA, CREM, CPT2, ADM, CRY1, PTGDS, KIDINS220, NUFIP2, LRRC7, DPP10, PTPRC, ATXN7, ERBB2, ATM, NF1, NFKB1, NGFR, NTRK1, NTRK3, OPRM1, MYT1, EPHA5, MTHFD1, EIF4EBP1, MYO5B, SPOCK3, PC, PFN1, PIK3CB, PIK3CD, DAPK3, CCHCR1, GPRC5B, ASCC1, AGA, DLX4, DLG2, IPO11, PLA1A, PDE4A, PDE4D, DGKH	ACACA, NAT8L, COL6A4P1, CADM1, SMUG1, IGF2, KCNH3, APP, APRT, ABCA4, HTR1E, HTR4, P4HTM, PRKCD, EAF2, ASH1L, NCAN, RELN, PSD, PRKCB, PPIA, TRIT1, TTC12, CYP2B6, SLC30A10, LXN, PEX2, RARA, CREM, ADM, BPIFA2, CRY1, PTGDS, CSE1L, KIDINS220, LRRC7, DPP10, HAMP, PTPRC, ATXN7, ATM, NF1, NFKB1, NGFR, NTRK1, MIR378A, OPRM1, MTHFD1, EGF, MYO5B, SPOCK3, PC, PIK3CB, PIK3CD, CNTN5, DAPK3, PON1, CCHCR1, GAL, DLX4, DLG2, PDE2A, PDE4A, PDE4D, OXER1, DGKH
**Curated Epilepsy Genes**	CACNA1A, CACNA1E, CACNA1G, CHD2, CHRNA4, CLN6, CLN8, CSTB, CTSD, DEPDC5, DNM1, DYNC1H1, DYRK1A, EEF1A2, FOLR1, FOXG1, GABRA1, GABRB3, GABRG2, GAMT, GATM, GNAO1, GRIN1, GRIN2A, GRIN2B, KCNA1, KCNA2, KCNB1, KCNC1, KCNJ10, KCNQ2, KCNQ3, KCNQ5, KCNT1, KCTD7, MEF2C, MFSD8, MTOR, NHLRC1, NPRL2, NPRL3, PIGT, PLCB1, PNKP, PNPO, PPT1, PRICKLE1, RELN, SCARB2, SCN1B, SCN2A, SCN8A, SLC12A5, SLC13A5, SLC25A22, SLC2A1, ST3GAL3, STX1B, STXBP1, SYNGAP1, SZT2, TBC1D24, TSC2, UBE3A, WWOX, ZEB2, ATP1A2, ATP1A3, CACNA1D, CERS1, CHRNA2, CNTNAP2, CPA6, EFHC1, GOSR2, HDAC4,	ASAH1, CACNA1A, CACNA1E, CACNA1G, CHD2, CHRNA4, CHRNB2, CLN3, CLN5, CLN6, CLN8, DEPDC5, DNM1, DYNC1H1, DYRK1A, FOXG1, GABRA1, GABRB3, GABRG2, GAMT, GATM, GNAO1, GRIN1, GRIN2A, GRIN2B, GRIN2D, HNRNPU, KCNA2, KCNB1, KCNC1, KCNJ10, KCNQ3, KCNQ5, KCNT1, KCTD7, LGI1, MEF2C, MTOR, NHLRC1, NPRL2, NPRL3, PIGT, PLCB1, PNPO, PRICKLE1, PRRT2, PURA, RELN, SCARB2, SCN1B, SCN8A, SLC12A5, SLC13A5, SLC25A22, SLC6A1, ST3GAL3, SYNGAP1, SZT2, TBC1D24, TPP1, TSC2, UBE3A, WWOX, ZEB2, ATP1A2, ATP1A3, CACNA1D, CNTNAP2, CPA6, EFHC1, GOSR2, HCN1, HDAC4, KCNMA1, POLG, QARS, RYR3, SIK1, CCDC30, CDADC1, CREB5, ELF3, EPX, ERC2,	ASAH1, CACNA1A, CACNA1E, CACNA1G, CHD2, CLN3, CLN5, CLN6, CLN8, DEPDC5, DYNC1H1, EEF1A2, EPM2A, FOLR1, GABRA1, GABRB3, GABRG2, GNAO1, GRIN1, GRIN2A, GRIN2B, GRIN2D, HNRNPU, KCNA1, KCNB1, KCNC1, KCNQ5, KCNT1, KCTD7, LGI1, MEF2C, NHLRC1, PIGT, PLCB1, PPT1, PRICKLE1, PURA, RELN, SCARB2, SCN1B, SCN8A, SLC12A5, SLC13A5, SLC25A22, SLC2A1, SLC6A1, SPTAN1, ST3GAL3, STXBP1, SYNGAP1, SZT2, TBC1D24, TPP1, TSC1, TSC2, UBE3A, WWOX, ZEB2, ATP1A2, CACNA1D, CNTNAP2, CPA6, GOSR2, HDAC4, IER3IP1, KCNMA1, POLG, RYR3, CCDC30, CDADC1, CREB5, EPX, ERC2, JUN, MAFG, MYT1L, PEX2, SKI

	IER3IP1, KCNMA1, POLG, QARS, RYR3, SIK1, CCDC30, CDADC1, CREB5, EPX, ERC2, JUN, MAFG, MAFK, MYT1L, PEX2, SKI, TRERF1, ZNF544	LRRC8B, MAFK, MYT1L, PEX2, PPIL1, SKI, TRERF1, ZNF544	
**DisGeNET Visuospatial Dysfunction**	POMT1, JRK, SLC2A1, PRKAR1B, DARS2, MLXIPL, MAPT, KCNC3, GABRG2, GABRB3, GABRA1, BPTF, ELN, CACNA1H	POMT1, JRK, PSMD12, DARS2, MLXIPL, MAPT, KCNC3, GABRG2, GABRB3, GABRA1, BPTF, ELN, CACNA1H	JRK, SLC2A1, PSMD12, PRKAR1B, DARS2, MLXIPL, MAPT, GABRG2, GABRB3, GABRA1, BPTF
**Positive Regulation Angiogenesis**	ACVRL1, ADD1, ADM, AGGF1, ALOX12, ANGPT4, ANGPTL4, ANXA3, AQP1, BRCA1, C3, C3AR1, C5, CCL11, CCL24, CD34, CIB1, CX3CR1, CXCR2, CYP1B1, CYSLTR2, DDAH1, EPHA1, ERAP1, ETS1, F3, FGF2, FOXC2, GATA2, GATA4, GREM1 HDAC7, HDAC9, HIF1A, HIPK1 HIPK2, HMOX1, HYAL1, SL1 ITGB2, KDR, MTDH, NOS3 NR2E1, PDCD6, PGF, PLCG1, PPP1R16B, PRKCB, PRKD1 PRKD2, PTGS2, PTK2B RAPGEF3, RLN2, RRAS RUNX1, SASH1, SEMA5A SERPINE1, SFRP2, SPHK1 TBXA2R, THBS1, TNFRSF1A TWIST1, UTS2R, WNT5A	ACVRL1, ADD1, ADM, ADM2, AGGF1, ALOX12, ANGPTL3, ANXA3, AQP1, BRCA1, BTG1, C3, C5, CCL11, CD34, CX3CL1, CX3CR1, CXCR2, CYP1B1, CYSLTR2, DDAH1, EPHA1, ERAP1, ETS1, F3, FGF1, FLT1, FOXC2, GATA4, GATA6, HDAC7, HDAC9, HIF1A, HMOX1, ISL1, ITGB2, KDR, MTDH, NODAL, NOS3, NR2E1, PDCD6, PLCG1, PPP1R16B, PRKCB, PRKD1, PRKD2, PTGIS, PTGS2, PTK2B, RAPGEF3, RUNX1, SASH1, SEMA5A, SFRP2, SPHK1, TBXA2R, TEK, TGFBR2, THBS1, TNFRSF1A, TWIST1, VEGFA, WNT5A	ACVRL1, ADD1, ADM, AGGF1, ALOX12, ANGPT4, ANGPTL4, AQP1, BRCA1, C3, CCL24, CD34, CIB1, CMA1, CX3CL1, CXCR2, CYP1B1, DDAH1, ETS1, F3, FGF1, FLT1, GATA2, GATA4, GATA6, GREM1, HDAC7, HDAC9, HIF1A, HIPK1, ISL1, KDR, NR2E1, PDCD6, PGF, PRKCB, PRKD1, PRKD2, PTGS2, PTK2B, RHOB, RRAS, RUNX1, SEMA5A, SERPINE1, SFRP2, SPHK1, TGFBR2, THBS1, TNFRSF1A, TWIST1
**ADSP Alzheimer's Associated**	SORT1, CR1, ADAM17, PRKD3 NCK2, BIN1, WDR12, INPP5D MME, IDUA, CLNK, RHOH ANKH, COX7C, RASGEF1C UNC5CL, TREM2, CD2AP HS3ST5, ICA1, TMEM106B JAZF1, EPDR1, SEC61G EPHA1, PTK2B, CLU, SHARPIN USP6NL, ANK3, TSPAN14, BLNK, PLEKHA1, EED, SORL1 TPCN1, FERMT2, SLC24A4 SPPL2A, APH1B, SNX1, DOC2A BCKDK, IL34, MAF, PLCG2 FOXF1, PRDM7, WDR81, SCIMP, MYO15A, GRN, ACE, ABCA7, SIGLEC11, LILRB2 RBCK1, CASS4, SLC2A4RG APP, APBB3, CASP7, MS4A6A PSEN1, PSEN2, ADAM10, RIN3 PICALM	SORT1, CR1, ADAM17, PRKD3, NCK2, BIN1, WDR12, INPP5D, MME, IDUA, CLNK, RHOH, ANKH, COX7C, TNIP1, RASGEF1C, UNC5CL, TREML2, CD2AP, HS3ST5, ICA1, JAZF1, EPDR1, EPHA1, CTSB, PTK2B, USP6NL, ANK3, TSPAN14, BLNK, MS4A4A, EED, SORL1, TPCN1, SLC24A4, SPPL2A, APH1B, SNX1, DOC2A, IL34, MAF, PLCG2, FOXF1, WDR81, MYO15A, ACE, ABCA7, SIGLEC11, RBCK1, CASS4, SLC2A4RG, APP, ADAMTS1, APBB3, CASP7, MS4A6A, PSEN1, PSEN2, ADAM10, RIN3	SORT1, CR1, PRKD3, NCK2, WDR12, INPP5D, MME, IDUA, ANKH, COX7C, TNIP1, RASGEF1C, TREML2, TMEM106B, JAZF1, EPDR1, SEC61G, CTSB, PTK2B, SHARPIN, USP6NL, ANK3, TSPAN14, BLNK, PLEKHA1, MS4A4A, SORL1, FERMT2, SNX1, DOC2A, IL34, PLCG2, FOXF1, WDR81, MYO15A, WNT3, RBCK1, CASS4, APP, CASP7, MS4A6A, PSEN1, ADAM10, RIN3, PICALM
**DisGeNET Executive Dysfunction**	BDNF, SLC6A3, SGCA, CHD2, CHD8, PSEN2, SNCA, CACNA1C, SPG11, ZBP1, CHD7, PNN, CSF1R, DRD2, TOR1A, ELK3, ICAM1, NGF, SFXN1	BDNF, SLC6A3, SGCA, CHD2, CHD8, PSEN2, SLC6A4, CACNA1C, SPG11, XPR1, CHD7, CSF1R, DRD2, ELK3, ICAM1, SFXN1	BDNF, COMT, SLC6A3, SGCA, CHD2, CHD8, CACNA1C, ZBP1, XPR1, CHD7, PNN, CSF1R, DRD2, ANKK1, ICAM1, NGF, SFXN1
**DisGeNET Social Anxiety**	TSPAN31, RGS2, ENOSF1, NANS, TBX4, CD5L, ZBP1, TSC2, HNF1B, OPRK1, NPY2R, ALB, ELK3, DNMT3B, DLG4, CYP2D6, KAT7, METAP2, KDM5B, SEPHS1, PDSS1, GGT1, MVD, MAP2, ARSI, NRG1, HDAC2, GRM7, GRN	SLC6A4, OXTR, TSPAN31, RGS2, NANS, CD5L, PPP1R9B, BEST1, TSC2, HNF1B, NPY2R, ALB, ELK3, DLG4, KAT7, KDM5B, SEPHS1, GGT1, NPY, GADD45B, MAP2, NRG1, HDAC2, GRM7	OXTR, GTF2I, CD69, RGS2, ENOSF1, OXT, TBX4, PPP1R9B, ZBP1, TSC2, HNF1B, OPRK1, NPY2R, DNMT1, KAT7, KDM5B, PDSS1, GGT1, GADD45B, MAP2, ARSI, GRM7

Significant genes with probe p-value <0.05
