# Supplementary figures and images for: DNA methylation differences stratified by normalized fetal/placental weight ratios suggest neurodevelopmental deficits in neonates with congenital heart disease
Source: PLoS One. 2025 Aug 6;20(8):e0317944. doi: 10.1371/journal.pone.0317944 (PMC12327636; doi:10.1371/journal.pone.0317944)

S1 Figure. Principal Component Analysis of Race

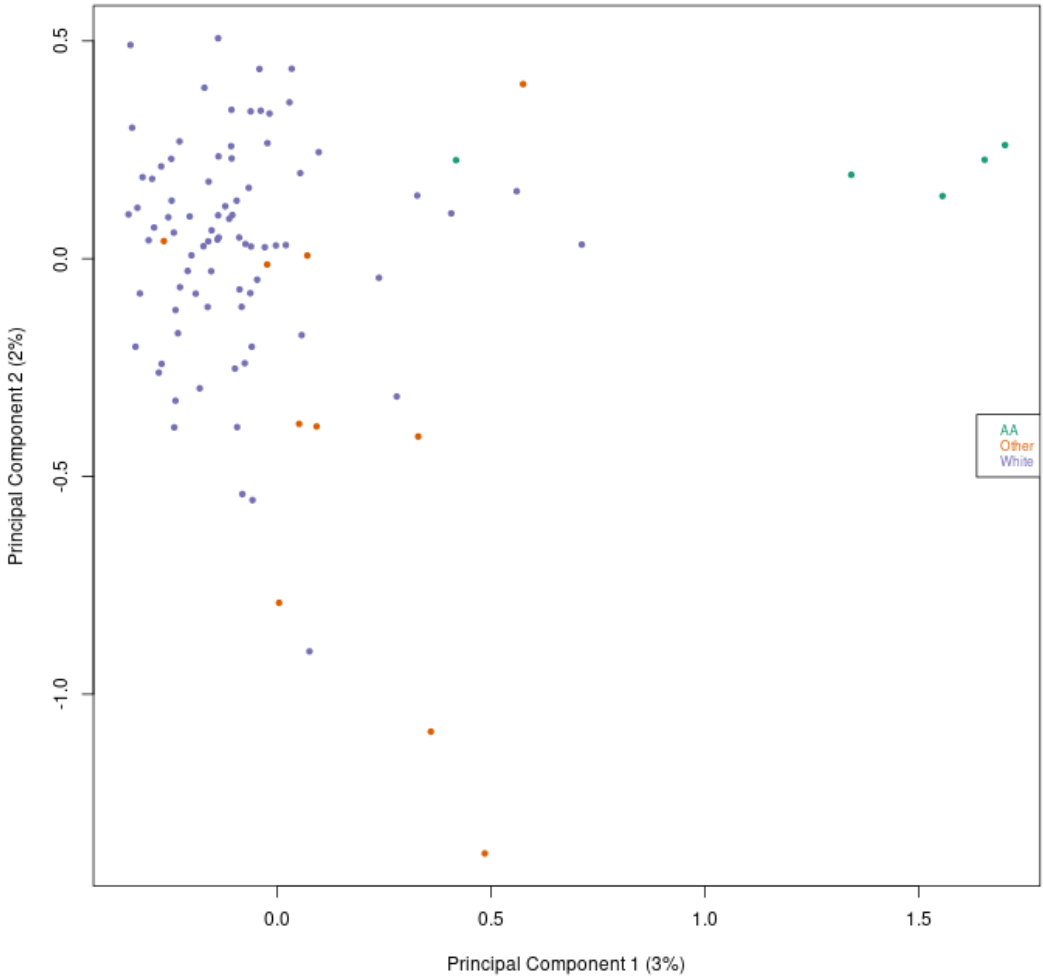

Supplement: S1 Fig — (PDF) [file pone.0317944.s002.pdf]

S2 Figure. Cell Type Composition in Umbilical Cord Blood and Postnatal DNA Blood Sample

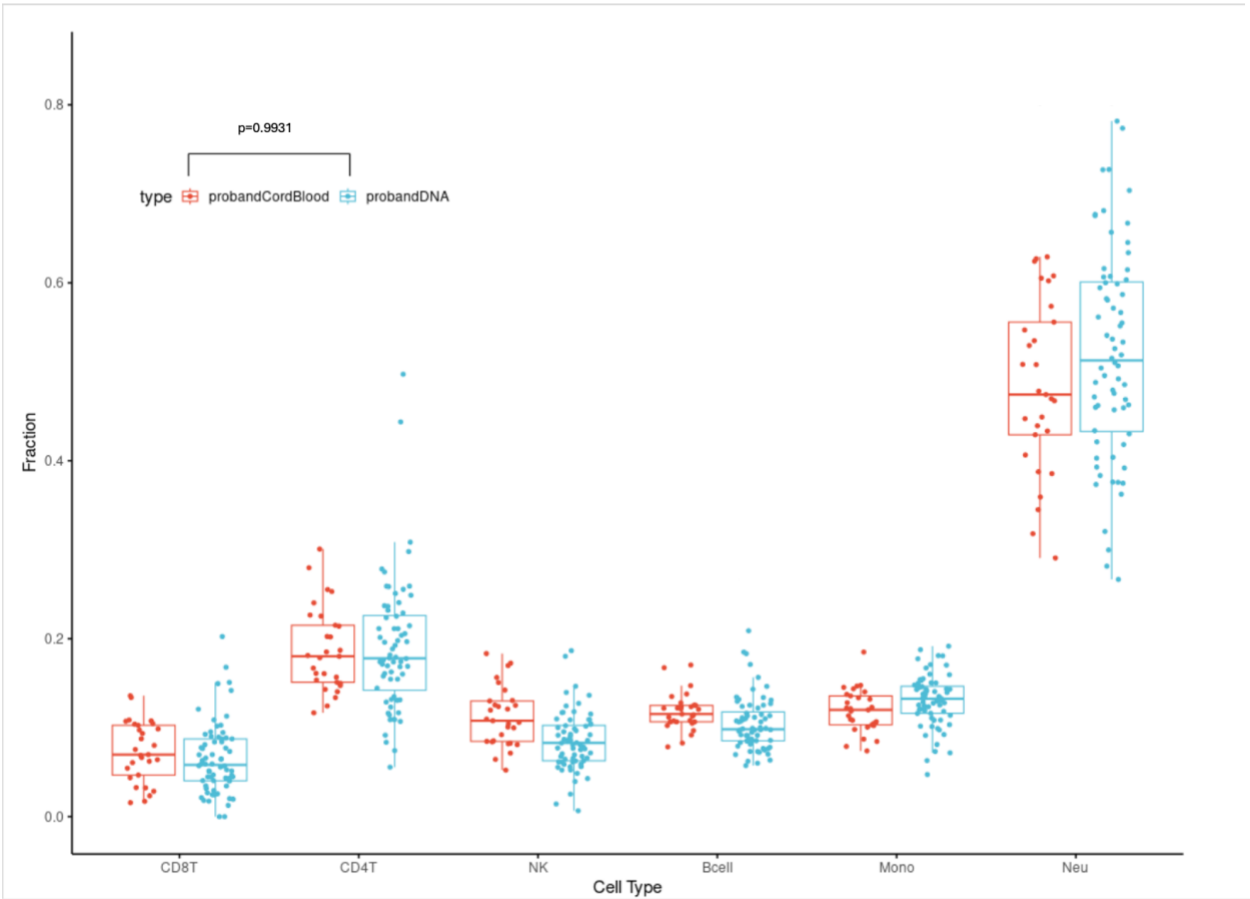

Supplement: S2 Fig — (PDF) [file pone.0317944.s003.pdf]

S3 Figure. BSID-III Stratified by Cardiac Lesion Type

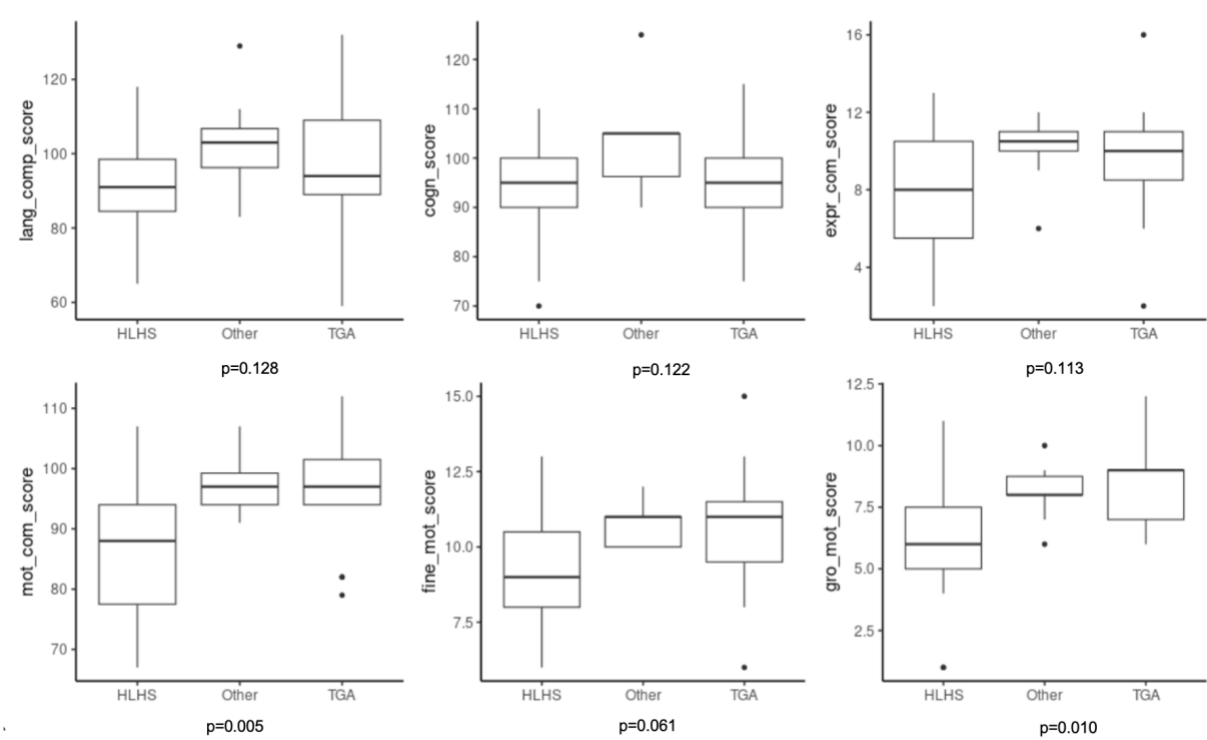

Supplement: S3 Fig — (PDF) [file pone.0317944.s004.pdf]
