# Supplementary material for: DNA methylation differences stratified by normalized fetal/placental weight ratios suggest neurodevelopmental deficits in neonates with congenital heart disease
Source: PLoS One. 2025 Aug 6;20(8):e0317944. doi: 10.1371/journal.pone.0317944 (PMC12327636; doi:10.1371/journal.pone.0317944)

S4 Figure. Association between total methylation rate and BSID-III (A full cohort, B female, C male) categorized as normal (85-115), mild impairment (0.5 SD below 85), severe impairment (≤2 SD below 85).

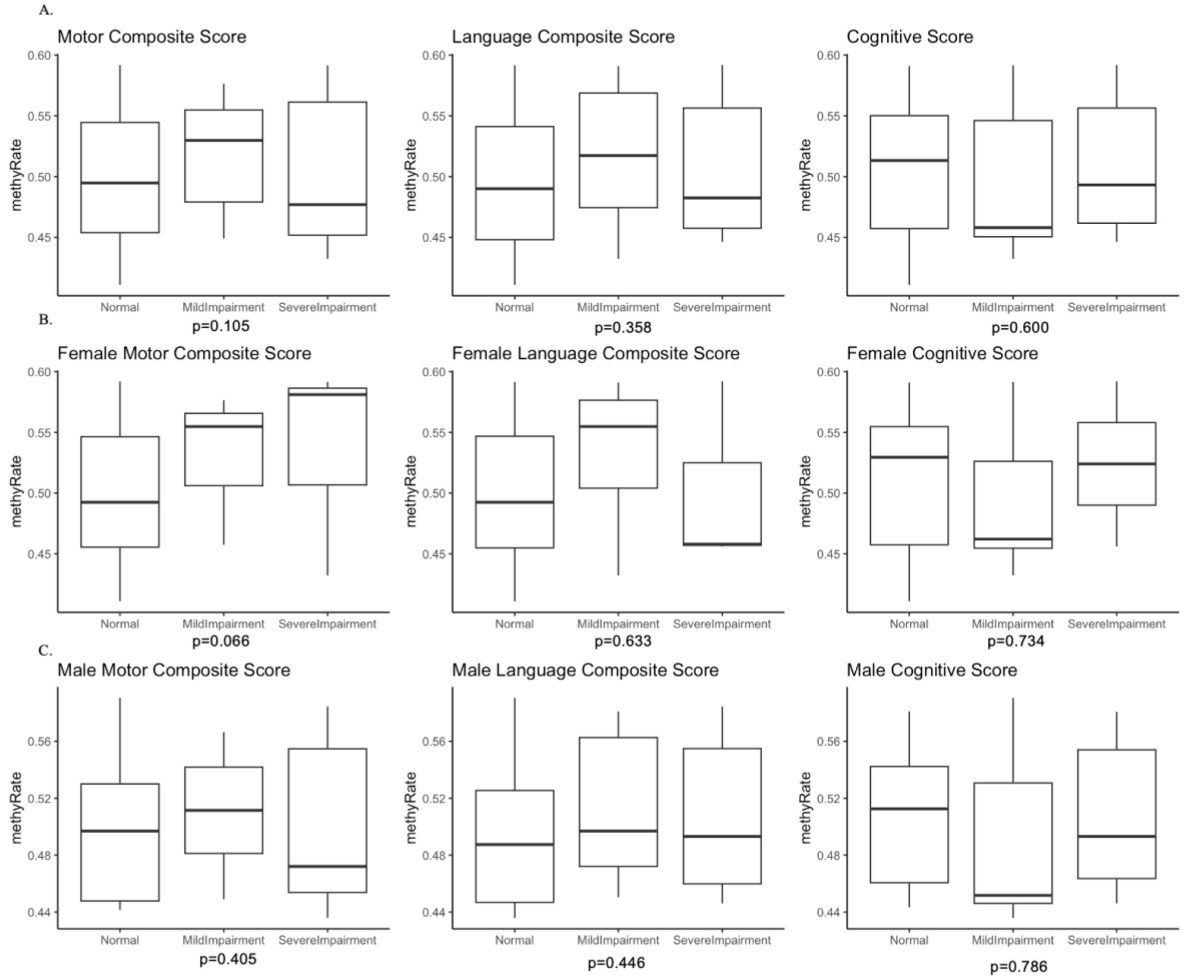

Supplement: S4 Fig — (PDF) [file pone.0317944.s005.pdf]
