# Supplementary material for: DNA methylation differences stratified by normalized fetal/placental weight ratios suggest neurodevelopmental deficits in neonates with congenital heart disease
Source: PLoS One. 2025 Aug 6;20(8):e0317944. doi: 10.1371/journal.pone.0317944 (PMC12327636; doi:10.1371/journal.pone.0317944)
Supplement: S1 File — (DOCX) [file pone.0317944.s009.docx]

Data Sharing Statement: There is an ethical restriction to public data sharing because the consent signed by parents requires individual study IRB approval for data sharing, including de-identified data. Interested investigators can contact the corresponding author for access to the data, at which time we will obtain appropriate IRB approval.
